# An ortho­rhom­bic polymorph of 5-[(4-methyl­phen­yl)diazen­yl]salicylaldehyde

**DOI:** 10.1107/S1600536809046868

**Published:** 2009-11-11

**Authors:** Tushar S. Basu Baul, Sajal Kundu, Hadi D. Arman, Edward R. T. Tiekink

**Affiliations:** a Department of Chemistry, North-Eastern Hill University, NEHU Permanent Campus, Umshing, Shillong 793 022, India; b Department of Chemistry, The University of Texas at San Antonio, One UTSA Circle, San Antonio, Texas 78249-0698, USA; c Department of Chemistry, University of Malaya, 50603 Kuala Lumpur, Malaysia

## Abstract

The title compound, C_14_H_12_N_2_O_2_, is an ortho­rhom­bic polymorph of the previously reported monoclinic form [Bakir *et al.* (2005 ▶). *Acta Cryst.* E**61**, o1611–o1613]. The dihedral angle between the aromatic rings is 4.32 (13)°. The mol­ecular structures of the two polymorphs, including short intra­molecular O—H⋯O hydrogen bonds between the the hydr­oxy and keto groups, are quite similar but their crystal packings are distinct. Unlike the monoclinic form, in which centrosymmetrically related hydr­oxy and keto groups form {⋯H⋯O}_2_ synthons *via* weak O—H⋯O contacts, leading to dimeric aggregates, in the ortho­rhom­bic form, the hydrogen bonding between these groups leads to the formation of supra­molecular chains orientated along the *a* axis.

## Related literature

For the structure of the monoclinic polymorph, see: Bakir *et al.* (2005 ▶). For background and motivation for the synthesis of the title compound, see: Basu Baul *et al.* (2005 ▶). For the synthesis, see: Sarma *et al.* (1993 ▶).
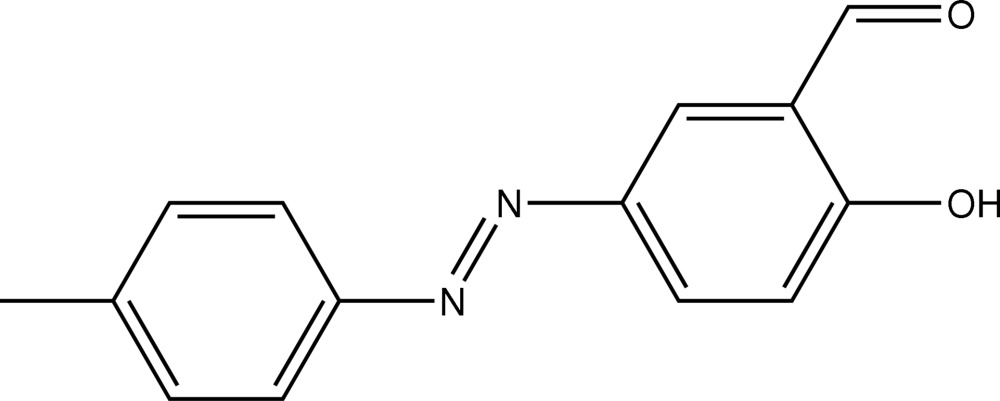


## Experimental

### 

#### Crystal data

C_14_H_12_N_2_O_2_
*M* *_r_* = 240.26
Orthorhombic, 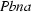

*a* = 6.016 (4) Å
*b* = 14.299 (9) Å
*c* = 26.878 (17) Å
*V* = 2312 (3) Å^3^
*Z* = 8
Mo *K*α radiation
μ = 0.09 mm^−1^
*T* = 98 K
0.25 × 0.18 × 0.15 mm

#### Data collection

Rigaku Saturn724 diffractometer
Absorption correction: none
12587 measured reflections
2032 independent reflections
1774 reflections with *I* > 2σ(*I*)
*R*_int_ = 0.082

#### Refinement

*R*[*F* ^2^ > 2σ(*F* ^2^)] = 0.081
*wR*(*F* ^2^) = 0.208
*S* = 1.14
2032 reflections
167 parameters
1 restraint
H-atom parameters constrained
Δρ_max_ = 0.40 e Å^−3^
Δρ_min_ = −0.32 e Å^−3^

### 

Data collection: *CrystalClear* (Rigaku/MSC, 2005 ▶); cell refinement: *CrystalClear*; data reduction: *CrystalClear*; program(s) used to solve structure: *SHELXS97* (Sheldrick, 2008 ▶); program(s) used to refine structure: *SHELXL97* (Sheldrick, 2008 ▶); molecular graphics: *DIAMOND* (Brandenburg, 2006 ▶); software used to prepare material for publication: *SHELXL97*.

## Supplementary Material

Crystal structure: contains datablocks global, I. DOI: 10.1107/S1600536809046868/hb5215sup1.cif

Structure factors: contains datablocks I. DOI: 10.1107/S1600536809046868/hb5215Isup2.hkl

Additional supplementary materials:  crystallographic information; 3D view; checkCIF report

## Figures and Tables

**Table 1:** Hydrogen-bond geometry (Å, °)

*D*—H⋯*A*	*D*—H	H⋯*A*	*D*⋯*A*	*D*—H⋯*A*
O1—H1o⋯O2	0.84	2.01	2.700 (3)	139
O1—H1o⋯O2^i^	0.84	2.44	3.008 (3)	125

Symmetry code: (i) 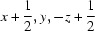.

**Fig. 1.**
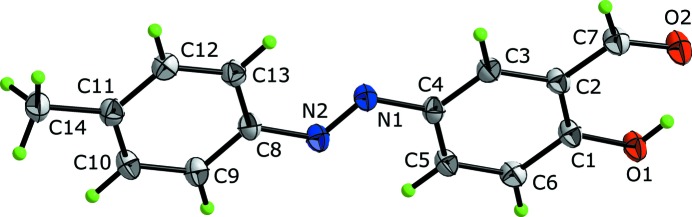
Molecular structure of (I) showing displacement ellipsoids at the 50% probability level.

**Fig. 2.**
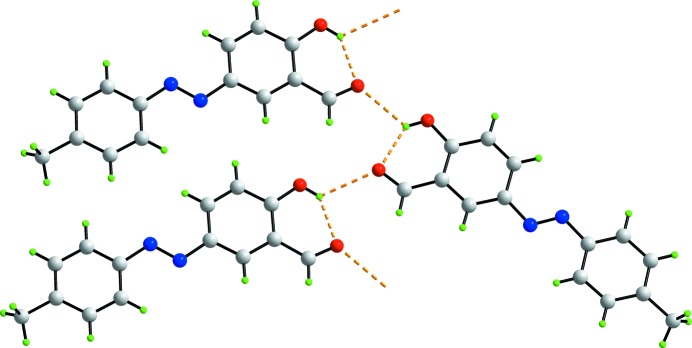
Supramolecular chain formation along the a axis in (I) mediated by O–H···O hydrogen bonds (orange dashed lines).

**Fig. 3.**
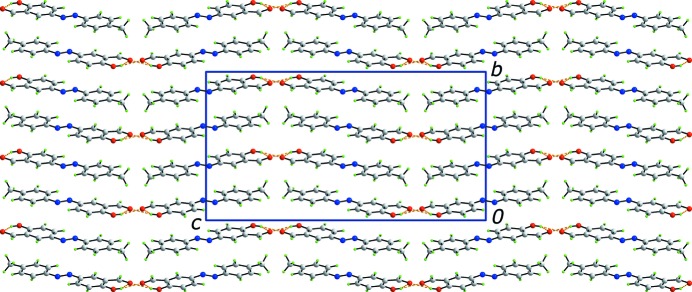
View in projection down the a axis of the crystal packing in (I), highlighting the stacking of supramolecular chains. The O–H···O hydrogen bonds are highlighted as orange dashed lines.

## supplementary crystallographic information

### Comment

The title compound, (I), was isolated during on-going studies into the biological activities of their organotin complexes (Basu Baul *et al.*, 2005). The structure of (I) represents an orthorhombic polymorph of a previously reported monoclinic form (Bakir *et al.*, 2005). The molecular structure of (I), Fig. 1, closely resembles that of the monoclinic form, with comparable geometric parameters being equal within experimental error. The overall molecule of (I) is planar as seen in the values of the C1/C2/C3/O2, C5/C4/N1/N2 and C9/C8/N2/N1 torsion angles of 0.9 (4), 0.7 (4) and -178.0 (2) °, respectively. In each of the polymorphs, a short intramolecular O–H···O hydrogen bond is formed between the hydroxyl and keto groups, Table 1. The key difference between the polymorphs rests with the nature of the weaker O–H···O intermolecular interactions formed between these groups. Thus, in the previously reported monoclinic form, the crystal structure comprises the packing of centrosymmetric dimers linked by a four-membered {···H···O}_2_ synthon. By contrast, in (I) the intermolecular hydrogen bonding between these groups leads to 1-D supramolecular chains aligned along the a direction, Table 1 and Fig. 2. The crystal structure comprises packing of these chains as illustrated in Fig. 3.

### Experimental

Compound (I) was prepared by reacting *p*-tolyldiazonium chloride with *o*-hydroxybenzaldehyde using a previously reported method (Sarma *et al.*, 1993). Orange crystals were obtained by slow evaporation of a methanol solution of (I); m.pt. 421–423 K.

### Refinement

Carbon-bound H-atoms were placed in calculated positions (C–H 0.95–0.98 Å) and were included in the refinement in the riding model approximation with *U*_iso_(H) set to 1.2–1.5*U*_eq_(C). The O–bound H-atom was located in a difference Fourier map and was refined with an O–H restraint of 0.840±0.001 Å, and with *U*_iso_(H) = 1.5*U*_eq_(O).

### Crystal data

**Table:**

C_14_H_12_N_2_O_2_	*F*(000) = 1008
*M**_r_* = 240.26	*D*_x_ = 1.380 Mg m^−^^3^
Orthorhombic, *P**b**n**a*	Mo *K*α radiation, λ = 0.71073 Å
Hall symbol: -P 2ac 2b	Cell parameters from 8423 reflections
*a* = 6.016 (4) Å	θ = 3.0–40.5°
*b* = 14.299 (9) Å	µ = 0.09 mm^−^^1^
*c* = 26.878 (17) Å	*T* = 98 K
*V* = 2312 (3) Å^3^	Prism, orange
*Z* = 8	0.25 × 0.18 × 0.15 mm

### Data collection

**Table:**

Rigaku Saturn724 diffractometer	1774 reflections with *I* > 2σ(*I*)
Radiation source: sealed tube	*R*_int_ = 0.082
graphite	θ_max_ = 25.0°, θ_min_ = 2.9°
Detector resolution: 28.5714 pixels mm^-1^	*h* = −5→7
ω scans	*k* = −17→17
12587 measured reflections	*l* = −31→31
2032 independent reflections	

### Refinement

**Table:**

Refinement on *F*^2^	Primary atom site location: structure-invariant direct methods
Least-squares matrix: full	Secondary atom site location: difference Fourier map
*R*[*F*^2^ > 2σ(*F*^2^)] = 0.081	Hydrogen site location: inferred from neighbouring sites
*wR*(*F*^2^) = 0.208	H-atom parameters constrained
*S* = 1.14	*w* = 1/[σ^2^(*F*_o_^2^) + (0.0971*P*)^2^ + 2.61*P*] where *P* = (*F*_o_^2^ + 2*F*_c_^2^)/3
2032 reflections	(Δ/σ)_max_ < 0.001
167 parameters	Δρ_max_ = 0.40 e Å^−^^3^
1 restraint	Δρ_min_ = −0.32 e Å^−^^3^

### Special details

**Table:**

Geometry. All s.u.'s (except the s.u. in the dihedral angle between two l.s. planes) are estimated using the full covariance matrix. The cell s.u.'s are taken into account individually in the estimation of s.u.'s in distances, angles and torsion angles; correlations between s.u.'s in cell parameters are only used when they are defined by crystal symmetry. An approximate (isotropic) treatment of cell s.u.'s is used for estimating s.u.'s involving l.s. planes.
Refinement. Refinement of *F*^2^ against ALL reflections. The weighted *R*-factor *wR* and goodness of fit *S* are based on *F*^2^, conventional *R*-factors *R* are based on *F*, with *F* set to zero for negative *F*^2^. The threshold expression of *F*^2^ > 2σ(*F*^2^) is used only for calculating *R*-factors(gt) *etc*. and is not relevant to the choice of reflections for refinement. *R*-factors based on *F*^2^ are statistically about twice as large as those based on *F*, and *R*- factors based on ALL data will be even larger.

### Fractional atomic coordinates and isotropic or equivalent isotropic displacement parameters (Å^2^)

**Table:**

	*x*	*y*	*z*	*U*_iso_*/*U*_eq_	
O1	0.5504 (3)	0.03566 (15)	0.16786 (7)	0.0290 (5)	
H1O	0.4957	0.0411	0.1965	0.043*	
O2	0.2044 (3)	0.07693 (15)	0.22794 (7)	0.0340 (6)	
N1	−0.0156 (4)	0.13491 (16)	0.01161 (7)	0.0245 (6)	
N2	0.0617 (4)	0.12120 (16)	−0.03149 (8)	0.0251 (6)	
C1	0.4095 (4)	0.0603 (2)	0.13084 (9)	0.0248 (6)	
C2	0.1931 (4)	0.09462 (19)	0.13980 (9)	0.0242 (6)	
C3	0.0583 (4)	0.12043 (19)	0.09890 (9)	0.0239 (6)	
H3	−0.0864	0.1446	0.1047	0.029*	
C4	0.1341 (4)	0.11101 (18)	0.05081 (9)	0.0226 (6)	
C5	0.3522 (5)	0.07589 (19)	0.04248 (9)	0.0250 (6)	
H5	0.4058	0.0698	0.0094	0.030*	
C6	0.4862 (4)	0.0508 (2)	0.08146 (9)	0.0252 (6)	
H6	0.6309	0.0269	0.0753	0.030*	
C7	0.1029 (5)	0.1000 (2)	0.18997 (10)	0.0285 (7)	
H7	−0.0444	0.1228	0.1938	0.034*	
C8	−0.0859 (4)	0.14457 (19)	−0.07115 (9)	0.0239 (6)	
C9	−0.0071 (5)	0.12717 (19)	−0.11890 (9)	0.0263 (7)	
H9	0.1367	0.1010	−0.1234	0.032*	
C10	−0.1380 (5)	0.1479 (2)	−0.16010 (10)	0.0276 (7)	
H10	−0.0824	0.1359	−0.1926	0.033*	
C11	−0.3501 (5)	0.18602 (19)	−0.15435 (9)	0.0265 (7)	
C12	−0.4272 (5)	0.20340 (19)	−0.10605 (9)	0.0262 (6)	
H12	−0.5709	0.2297	−0.1015	0.031*	
C13	−0.2985 (5)	0.18306 (19)	−0.06474 (10)	0.0256 (6)	
H13	−0.3537	0.1951	−0.0323	0.031*	
C14	−0.4928 (5)	0.2058 (2)	−0.19935 (10)	0.0341 (7)	
H14A	−0.4410	0.2631	−0.2156	0.051*	
H14B	−0.6477	0.2137	−0.1889	0.051*	
H14C	−0.4823	0.1533	−0.2227	0.051*	

### Atomic displacement parameters (Å^2^)

**Table:**

	*U*^11^	*U*^22^	*U*^33^	*U*^12^	*U*^13^	*U*^23^
O1	0.0230 (11)	0.0436 (13)	0.0203 (10)	0.0040 (9)	−0.0026 (7)	−0.0010 (8)
O2	0.0260 (11)	0.0533 (14)	0.0227 (10)	0.0007 (9)	−0.0016 (8)	0.0003 (9)
N1	0.0246 (12)	0.0289 (13)	0.0202 (12)	0.0002 (10)	−0.0018 (9)	0.0010 (9)
N2	0.0238 (12)	0.0293 (13)	0.0221 (12)	−0.0007 (10)	−0.0029 (10)	0.0001 (9)
C1	0.0214 (14)	0.0306 (15)	0.0224 (13)	−0.0030 (11)	−0.0023 (10)	−0.0018 (11)
C2	0.0223 (13)	0.0280 (15)	0.0222 (13)	−0.0001 (11)	0.0021 (11)	−0.0006 (10)
C3	0.0197 (13)	0.0282 (14)	0.0239 (14)	0.0017 (11)	−0.0011 (11)	−0.0012 (10)
C4	0.0212 (14)	0.0248 (14)	0.0217 (13)	−0.0024 (10)	−0.0033 (11)	0.0005 (10)
C5	0.0242 (14)	0.0306 (15)	0.0203 (12)	−0.0003 (11)	0.0014 (11)	−0.0021 (11)
C6	0.0191 (13)	0.0312 (15)	0.0252 (13)	0.0002 (11)	0.0008 (11)	−0.0021 (11)
C7	0.0240 (14)	0.0362 (16)	0.0255 (14)	0.0008 (12)	0.0011 (12)	−0.0004 (11)
C8	0.0231 (14)	0.0267 (15)	0.0219 (13)	−0.0015 (11)	−0.0026 (11)	0.0001 (10)
C9	0.0241 (14)	0.0298 (15)	0.0251 (14)	−0.0025 (11)	−0.0012 (11)	0.0007 (11)
C10	0.0273 (14)	0.0350 (16)	0.0204 (13)	−0.0023 (12)	0.0020 (11)	−0.0014 (11)
C11	0.0279 (15)	0.0258 (15)	0.0258 (14)	−0.0033 (11)	−0.0034 (12)	0.0009 (11)
C12	0.0223 (14)	0.0277 (15)	0.0285 (14)	−0.0005 (11)	−0.0004 (11)	0.0007 (11)
C13	0.0278 (15)	0.0256 (15)	0.0234 (13)	−0.0016 (11)	0.0002 (11)	0.0009 (10)
C14	0.0369 (16)	0.0407 (18)	0.0248 (14)	0.0037 (14)	−0.0051 (12)	0.0033 (12)

### Geometric parameters (Å, °)

**Table:**

O1—C1	1.353 (3)	C7—H7	0.9500
O1—H1O	0.8400	C8—C9	1.391 (4)
O2—C7	1.234 (3)	C8—C13	1.403 (4)
N1—N2	1.264 (3)	C9—C10	1.391 (4)
N1—C4	1.428 (3)	C9—H9	0.9500
N2—C8	1.427 (3)	C10—C11	1.396 (4)
C1—C6	1.411 (4)	C10—H10	0.9500
C1—C2	1.413 (4)	C11—C12	1.401 (4)
C2—C3	1.415 (4)	C11—C14	1.510 (4)
C2—C7	1.455 (4)	C12—C13	1.385 (4)
C3—C4	1.377 (4)	C12—H12	0.9500
C3—H3	0.9500	C13—H13	0.9500
C4—C5	1.423 (4)	C14—H14A	0.9800
C5—C6	1.370 (4)	C14—H14B	0.9800
C5—H5	0.9500	C14—H14C	0.9800
C6—H6	0.9500		
			
C1—O1—H1O	113.8	C9—C8—C13	119.6 (2)
N2—N1—C4	114.0 (2)	C9—C8—N2	115.8 (2)
N1—N2—C8	114.8 (2)	C13—C8—N2	124.6 (2)
O1—C1—C6	117.5 (2)	C8—C9—C10	120.2 (3)
O1—C1—C2	122.8 (2)	C8—C9—H9	119.9
C6—C1—C2	119.7 (2)	C10—C9—H9	119.9
C1—C2—C3	119.1 (2)	C9—C10—C11	120.8 (2)
C1—C2—C7	121.3 (2)	C9—C10—H10	119.6
C3—C2—C7	119.5 (2)	C11—C10—H10	119.6
C4—C3—C2	120.9 (2)	C10—C11—C12	118.3 (2)
C4—C3—H3	119.5	C10—C11—C14	120.3 (2)
C2—C3—H3	119.5	C12—C11—C14	121.4 (3)
C3—C4—C5	119.2 (2)	C13—C12—C11	121.4 (3)
C3—C4—N1	117.4 (2)	C13—C12—H12	119.3
C5—C4—N1	123.4 (2)	C11—C12—H12	119.3
C6—C5—C4	121.0 (2)	C12—C13—C8	119.6 (2)
C6—C5—H5	119.5	C12—C13—H13	120.2
C4—C5—H5	119.5	C8—C13—H13	120.2
C5—C6—C1	120.1 (2)	C11—C14—H14A	109.5
C5—C6—H6	119.9	C11—C14—H14B	109.5
C1—C6—H6	119.9	H14A—C14—H14B	109.5
O2—C7—C2	124.6 (3)	C11—C14—H14C	109.5
O2—C7—H7	117.7	H14A—C14—H14C	109.5
C2—C7—H7	117.7	H14B—C14—H14C	109.5
			
C4—N1—N2—C8	179.9 (2)	C2—C1—C6—C5	0.8 (4)
O1—C1—C2—C3	178.8 (2)	C1—C2—C7—O2	0.9 (5)
C6—C1—C2—C3	−1.1 (4)	C3—C2—C7—O2	178.1 (3)
O1—C1—C2—C7	−4.0 (4)	N1—N2—C8—C9	−178.0 (2)
C6—C1—C2—C7	176.1 (3)	N1—N2—C8—C13	2.2 (4)
C1—C2—C3—C4	1.1 (4)	C13—C8—C9—C10	0.1 (4)
C7—C2—C3—C4	−176.2 (3)	N2—C8—C9—C10	−179.8 (2)
C2—C3—C4—C5	−0.8 (4)	C8—C9—C10—C11	−0.2 (4)
C2—C3—C4—N1	177.4 (2)	C9—C10—C11—C12	0.3 (4)
N2—N1—C4—C3	−177.4 (2)	C9—C10—C11—C14	−178.4 (3)
N2—N1—C4—C5	0.7 (4)	C10—C11—C12—C13	−0.4 (4)
C3—C4—C5—C6	0.6 (4)	C14—C11—C12—C13	178.3 (3)
N1—C4—C5—C6	−177.6 (3)	C11—C12—C13—C8	0.2 (4)
C4—C5—C6—C1	−0.6 (4)	C9—C8—C13—C12	−0.1 (4)
O1—C1—C6—C5	−179.0 (2)	N2—C8—C13—C12	179.7 (2)

### Hydrogen-bond geometry (Å, °)

**Table:**

*D*—H···*A*	*D*—H	H···*A*	*D*···*A*	*D*—H···*A*
O1—H1o···O2	0.84	2.01	2.700 (3)	139
O1—H1o···O2^i^	0.84	2.44	3.008 (3)	125

Symmetry codes: (i) *x*+1/2, *y*, −*z*+1/2.
